# Lipomatous hemangiopericytoma (adipocytic variant of solitary fibrous tumor) of the parotid gland: A case report and review of the literature

**DOI:** 10.3892/ol.2013.1538

**Published:** 2013-08-21

**Authors:** DAN CHEN, JIZE XUAN, MEIYU SUN, HONGWEI GUAN

**Affiliations:** 1 Department of Pathology, The First Affiliated Hospital of Dalian Medical University, Dalian, Liaoning 116001, P.R. China; 2 Department of Radiology, The First Affiliated Hospital of Dalian Medical University, Dalian, Liaoning 116001, P.R. China

**Keywords:** parotid gland, lipomatous hemangiopericytoma, hemangiopericytoma, solitary fibrous tumor, immunohistochemistry

## Abstract

The current study presents the first case of a lipomatous hemangiopericytoma (LHPC) developing in the parotid gland in a 33-year-old male. The patient presented with a 4-year history of a progressively growing painless and fixed mass in the left paratid gland region. The patient underwent radical parotidectomy and was followed-up for 12 months without any evidence of metastasis or recurrence. LHPC, a controversial rare HPC variant, is histologically characterized by a varying admixture of hemangiopericytomatous vasculature and the presence of mature adipocytes. To date, 51 cases of LHPC have been documented in the literature. Although the boundary between HPC and solitary fibrous tumors (SFTs) has become increasingly blurred, neither of these variant growth patterns has previously been recognized in the parotid gland.

## Introduction

Lipomatous hemangiopericytoma (LHPC) is an uncommon, slow-growing, almost non-recurring, non-metastasizing mesenchymal neoplasm composed of mature adipocytes and HPC-like areas (1). The majority of these tumors are located in the deep soft tissue (2). Other commonly affected sites include the thigh, retroperitoneum and the orbit (3). Grossly, LHPC generally presents as a well-circumscribed, often non-encapsulated, medium-sized mass with alternating areas of white/yellow tumor tissue in the cut surface (2). Microscopically, these tumors characteristically show a varying combination of patternless cellular areas composed of round to spindle-shaped cells, hemangiopericytoma-like vascular areas made up of small- to medium-sized thin-walled branched vessles, and lipomatous areas made up of mature adipocytes (2–6). Long-term follow-up is recommended (3,5). To date, 51 cases of LHPC have been documented in the published literature (2–6). However, there have been no reports of parotid gland LHPC. The current study presents the clinicopathological features of the first case of parotid gland LHPC in a 33-year-old male, along with a review of the literature. Written informed consent was obtained from the patient.

## Case report

**Figure 1:**
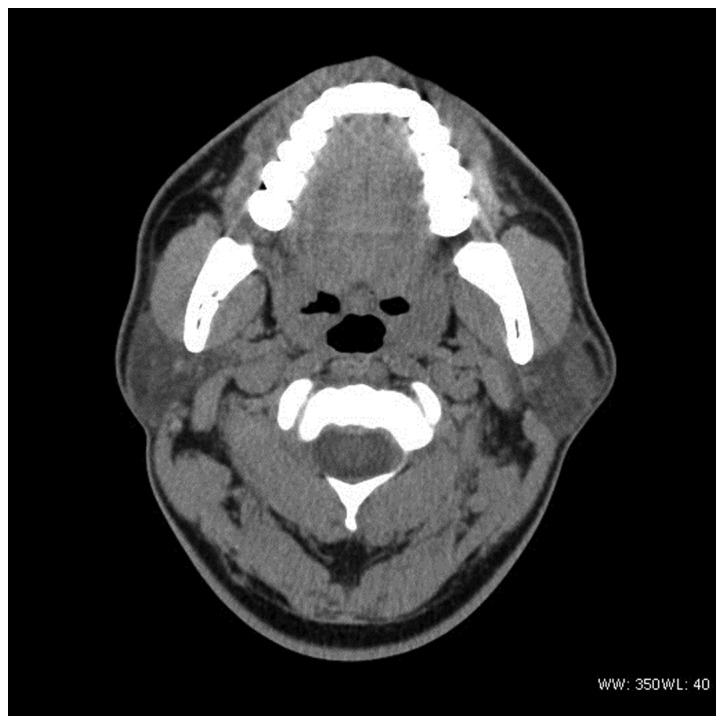
CT plain scan showing an oval, heterogeneous, well-defined mass in the left parotid gland.

**Figure 2:**
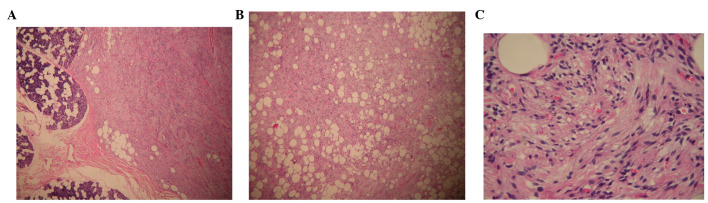
(A) Low-power histological appearance showing a well demarcated lesion. Hematoxylin and eosin (HE), original magnification, ×40. (B) A patternless architecture of short spindle cells surrounding the thin-walled branching vessels intermingled with mature adipocytes (HE; original magnification, ×40). (C) Non-lipomatous tumor cells with a weakly eosinophilic cytoplasm and spindle-shaped nuclei with finely granular chromatin (HE; original magnification, ×200).

**Figure 3:**
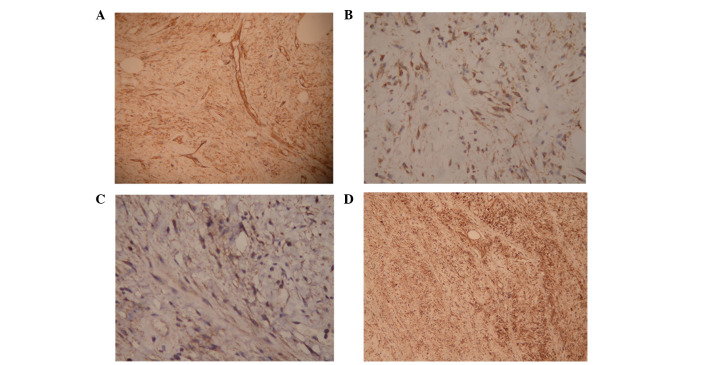
Immunohistochemical stains showing the positivity of tumor cells for (A) CD34, (B) Bcl-2, (C) CD99 (immunoperoxidase stains, original magnification, ×600) and (D) vimentin (immunoperoxidase stains, original magnification, ×100).

A 33-year-old male presented with a four-year history of a progressively growing, painless and fixed mass in the left parotid gland region. The patient did not have any associated headaches, facial weakness or numbness, or impairments in speech or swallowing. A head and neck CT scan showed a 19.0×13.2-mm oval heterogeneous mass within the left parotid gland (Fig. 1). A radical parotidectomy was performed and an irregular, well-circumscribed mass compromising the entire gland was observed during the surgery. The well-defined mass had a solid and tan/white cut surface. The histopathological study identified a well-circumscribed lesion composed of cellular nodules with the classic appearance of an HPC area admixed with clusters and lobules of mature adipocytes. The ill-defined tumor cells had a weakly eosinophilic cytoplasm and spindle-shaped nuclei, with occasional small nucleoli. Nuclei atypia and mitoses were absent, and no cellular atypia, necrosis or vascular invasion was observed (Fig. 2). Immunohistochemistry showed that the tumor cells were diffusely positive for CD34, CD99, Bcl-2 and vimentin (Fig. 3). Stains for cytokeratin, EMA, HMB45, S100, CD117 and p63 were negative. The patient was managed with surgery without any further treatment and had an uneventful clinical course post-operatively after a follow-up of 12 months.

## Discussion

LHPC was first proposed as a unique HPC variant in 1995 by Nielsen *et al*(4) for tumors composed of an admixture of hemangiopericytomatous areas and mature adipose tissue. HPC is a controversial entity. In addition to the lack of a definite immunophenotype, the relative non-specificity of the characteristic branching capillary pattern and cytological features of the constituent cells has led to uncertainty and a lack of consensus concerning this subgroup of tumors. Due to the major histological overlap between solitary fibrous tumors (SFTs) and HPC, and the lack of evident criteria to judge whether a lesion should be called an SFT or HPC, pathologists have gradually abandoned the term HPC in favor of the term SFT, so that the majority of lesions that were called HPCs 15 years ago now tend to be called SFTs (7).

Guillou *et al*(2) reviewed 100 extrapleural SFTs and encountered 13 neoplasms that characteristically contained islands of mature fatty tissue, and suggested that LHPC does not correspond to a well-defined entity, but rather is representative of a fat-containing variant of SFT. According to the 2002 edition of the World Health Organization (WHO) Classification of Tumors of Soft Tissue and Bone (1), there is an overlap between SFT and both LHPC and giant cell angiofibroma. However, the term LHPC is reserved for a variant of HPC. To date, neither of these variant growth patterns has yet been recognized in the parotid gland. All of the SFTs reported in the parotid gland have been of the ‘fibrous variant’ (8).

To date, a total of 52 pathologically confirmed cases of LHPC have been reported, including that of the present patient (2–6). These patients consisted of 31 males and 21 females, ranging in age from 21 to 79 years, with average and median ages of 50.96 and 51 years, respectively. LHPC generally presents as a well-circumscribed, often non-encapsulated, medium-sized mass with alternating areas of whitish and yellowish tumor tissue in the cut surface (2). The histological appearance of LHPC consists of a varying combination of patternless cellular areas composed of round to spindle-shaped cells, an HPC-like vasculature made of small- to medium-sized thin-walled branched vessels and lipomatous areas consisting of mature adipocytes. Immunohistochemistry has shown that non-adipocytic tumor cells are consistently positive for CD99 and, less frequently, for CD34 (75%) and Bcl-2 (60%) (1). Ultrastructural features are non-specific in HPC and the majority of the lesions reported as HPC have only shown undifferentiated spindle cell or fibroblastic features (2–6). Convincing evidence of true pericytic differentiation has not been observed (1).

Considering the location and histological appearance of HPC, the main differential diagnoses considered for this type of tumor should include pleomorphic adenoma, myoepithelioma, schwannoma, neurofibroma and angiomyolipoma. Glial fibrillary acidic protein (GFAP) is often positively expressed in pleomorphic adenoma, and would be of additional assistance in excluding this diagnosis. S100, GFAP and p63 are positively expressed in myoepithelioma. Schwannoma and neurofibroma are strongly reactive with S100 protein, while lacking CD34. Angiomyolipoma reacts with HMB45, Melan-A, tyrosinase and S100 protein.

The most common treatment for LHPC is complete local surgical excision. Although no LHPC patients developed recurrence during the follow-up interval and even though all patients were without disease, certain authors have recommended the use of a long-term follow-up (3,5). In conclusion, the present study describes the first case of a parotid gland LHPC. The clinicopathological features of this case were similar to those of normal LHPC. More cases of LHPC should be studied to provide further understanding of the behavior of this rare tumor.

## References

[1] Guillou L, Fletcher JA, Fletcher CDM, Mandahl N, Fletcher CDM, Unni KK, Mertens F (2002). Extrapleural solitary fibrous tumour and hemangiopericytoma. Pathology and Genetics of Tumours of Soft Tissue and Bone.

[2] Guillou L, Gebhard S, Coindre JM (2000). Lipomatous hemangiopericytoma: a fat-containing variant of solitary fibrous tumor? Clinicopathologic, immunohistochemical, and ultrastructural analysis of a series in favor of a unifying concept. Hum Pathol.

[3] Jing HB, Meng QD, Tai YH (2011). Lipomatous hemangiopericytoma of the stomach: a case report and a review of literature. World J Gastroenterol.

[4] Nielsen GP, Dickersin GR, Provenzal JM, Rosenberg AE (1995). Lipomatous hemangiopericytoma. A histologic, ultrastructural and immunohistochemical study of a unique variant of hemangiopericytoma. Am J Surg Pathol.

[5] Davies PE, Davis GJ, Dodd T, Selva D (2002). Orbital lipomatous haemangiopericytoma: an unusual variant. Clin Experiment Ophthalmol.

[6] Barazani Y, Tareen B (2012). Rare case of paratesticular solitary fibrous tumour (lipomatous hemangiopericytoma). Can Urol Assoc J.

[7] Gengler C, Guillou L (2006). Solitary fibrous tumour and haemangiopericytoma: evolution of a concept. Histopathology.

[8] Bauer JL, Miklos AZ, Thompson LD (2012). Parotid gland solitary fibrous tumor: a case report and clinicopathologic review of 22 cases from the literature. Head Neck Pathol.

